# Prolactin Induces Apoptosis of Lactotropes in Female Rodents

**DOI:** 10.1371/journal.pone.0097383

**Published:** 2014-05-23

**Authors:** Jimena Ferraris, Sandra Zárate, Gabriela Jaita, Florence Boutillon, Marie Bernadet, Julien Auffret, Adriana Seilicovich, Nadine Binart, Vincent Goffin, Daniel Pisera

**Affiliations:** 1 Instituto de Investigaciones Biomédicas, UBA-CONICET, Paraguay, Ciudad Autónoma de Buenos Aires, Argentina; 2 INSERM, Unit 1151, Institut Necker Enfants Malades (INEM), Team “PRL/GH Pathophysiology”, University Paris Descartes, Sorbonne Paris Cité, Faculty of Medicine, Bâtiment LERICHE, 14 Rue Maria Helena Vieira Da Silva, CS61431, 75993 Paris Cedex 14, France; 3 INSERM U693 and Université Paris-Sud, Faculté de Médecine Paris-Sud, UMR-S693, Le Kremlin-Bicêtre, F-94276 France; University of Michigan School of Medicine, United States of America

## Abstract

Anterior pituitary cell turnover occurring during female sexual cycle is a poorly understood process that involves complex regulation of cell proliferation and apoptosis by multiple hormones. In rats, the prolactin (PRL) surge that occurs at proestrus coincides with the highest apoptotic rate. Since anterior pituitary cells express the prolactin receptor (PRLR), we aimed to address the actual role of PRL in the regulation of pituitary cell turnover in cycling females. We showed that acute hyperprolactinemia induced in ovariectomized rats using PRL injection or dopamine antagonist treatment rapidly increased apoptosis and decreased proliferation specifically of PRL producing cells (lactotropes), suggesting a direct regulation of these cell responses by PRL. To demonstrate that apoptosis naturally occurring at proestrus was regulated by transient elevation of endogenous PRL levels, we used PRLR-deficient female mice (PRLRKO) in which PRL signaling is totally abolished. According to our hypothesis, no increase in lactotrope apoptotic rate was observed at proestrus, which likely contributes to pituitary tumorigenesis observed in these animals. To decipher the molecular mechanisms underlying PRL effects, we explored the isoform-specific pattern of PRLR expression in cycling wild type females. This analysis revealed dramatic changes of long *versus* short PRLR ratio during the estrous cycle, which is particularly relevant since these isoforms exhibit distinct signaling properties. This pattern was markedly altered in a model of chronic PRLR signaling blockade involving transgenic mice expressing a pure PRLR antagonist (TG^Δ1–9-G129R-hPRL^), providing evidence that PRL regulates the expression of its own receptor in an isoform-specific manner. Taken together, these results demonstrate that i) the PRL surge occurring during proestrus is a major proapoptotic signal for lactotropes, and ii) partial or total deficiencies in PRLR signaling in the anterior pituitary may result in pituitary hyperplasia and eventual prolactinoma development, as observed in TG^Δ1–9-G129R-hPRL^ and PRLRKO mice, respectively.

## Introduction

Prolactin (PRL) is a hormone secreted mainly by lactotropes in the anterior pituitary gland. This hormone is involved in several physiological functions, including mammopoiesis, lactogenesis and reproduction [1] although it has also been implicated in the development of various peripheral tumors. In breast and prostate cancers, where local PRL production has been demonstrated, its proliferative potential via an autocrine/paracrine mechanisms has been proposed to contribute to tumor development and progression [2], [3], [4], [5], [6], [7].

Prolactinomas are benign tumors that constitute approximately one third of pituitary tumors [2]. One of the main characteristics of prolactinomas is that they rarely undergo malignant transformation or local invasiveness [2]. It has been proposed that prolactinomas have a monoclonal origin and that alterations in cell cycle regulation lead to expansion of an original mutated cell [8], [9]. Although several oncogenes are expressed in these adenomas, and various mutations have been associated with familial cases of anterior pituitary tumors, the mechanisms leading to sporadic adenoma formation, the most frequent presentation of the pathology in this gland, are currently unknown [2], [8], [9]. Considering that the anterior pituitary is a gland with considerable plasticity [10], alterations in the mechanisms that physiologically regulate anterior pituitary cell turnover can be involved in the pathogenesis of pituitary tumors. Since all endocrine pituitary cells, including lactotropes, express prolactin receptors (PRLR) [11], [12], PRL is assumed to participate in the regulation of anterior pituitary functions including tissue homeostasis. Hence, alterations of PRLR signaling may play a role in anterior pituitary tumor development.

According to the effects described for PRL in the majority of its target tissues [6], [13], [14], [15], [16], [17], [18], it was initially proposed that this hormone may exert trophic action on anterior pituitary cells [19], [20], [21], [22]. However, studies using PRLR knockout (PRLRKO) mice subsequently showed that PRL actually exerts an opposite effect on lactotropes, since these mice develop pituitary adenomas [23]. Using a specific PRLR antagonist able to partially block PRLR signaling in biological systems where both the ligand and the receptor are expressed, we recently demonstrated that unlike what happens in most other tissues, PRL induced apoptosis and reduced proliferation of anterior pituitary cells from male rats, acting through an autocrine/paracrine mechanism [24].

In females, however, regulation of pituitary homeostasis is a more complex process that remains uncharacterized. The anterior pituitary gland of female rodents undergoes constant remodeling during each estrous cycle. Furthermore, under specific conditions such as pregnancy and lactation, it also responds to particular physiological demands [10], [25], [26]. Anterior pituitary cell turnover is about 3% per day in female rats [10]. During each estrous cycle, a peak of proliferation occurs specifically at estrus [27], [28], [29] whereas the highest rate of apoptosis is observed at proestrus [30], [31]. This cell turnover is a tightly regulated process in which several factors, e.g. estradiol [25], [32], dopamine [33], [34], and 16 kDa PRL [35], were demonstrated to participate. Interestingly, during the afternoon of proestrus, i.e. when the rate of apoptosis is the highest, there is a concomitant peak of serum PRL in response to high circulating levels of estrogens [36]. We hypothesize that the proestrus surge of PRL release participates in anterior pituitary cell renewal that occurs during the estrous cycle.

The PRLR is expressed as different isoforms generated by alternative splicing. They include one long (PRLR_long_) and one short (PRLR_short_) isoform in rats and one long and three short (S1, S2 and S3) isoforms in mice [2]. The long and short isoforms differ in the polypeptide chain of the intracellular domain; hence, they are all able to bind PRL equally but exhibit different abilities to trigger the canonical PRLR intracellular signaling pathways [4], [37], [38]. Although PRLR isoforms are usually co–expressed in the same tissue [38], [39], one of the isoforms often predominates over the others, depending on the tissue and the physiological context [37], [39]. For example, in the mouse ovary, PRLR_long_ is the most abundantly expressed isoform, followed by S2 and S3 PRLR_short_ isoforms [40]. Furthermore, the ratio of PRLR_long_ versus PRLR_short_ expression varies during the estrous cycle, as showed in the rat ovary [41]. Transgenic mice expressing only the PRLR_short_ exhibit impaired follicular and corpora lutea development [42], [43] whereas mice expressing only the endogenous PRLR_long_ present normal follicular but abnormal corpus luteum development. This suggests that the expression of both PRLR isoforms is necessary for physiological completion of this process [41].

Regarding the pituitary, expression of long and short isoforms has been reported in the rat, the PRLR_long_ being the most abundant. In the male mouse pituitary, the predominant form is also the PRLR_long_ [24]. In the female mouse pituitary, PRLR expression was studied in prepuberal mice, where the short isoform was found to be predominant [44]. In mature female mice, expression of the four PRLR isoforms has been reported [45]. However, data on PRLR expression in cycling female mice are lacking, which makes delineating the actual involvement of PRLR signaling in pituitary homeostasis during each estrous cycle elusive. Indeed, PRL may autoregulate its actions on the anterior pituitary by modulating its own expression, its own secretion [46] and/or the isoform-specific expression of its receptor, as described in a variety of other tissues [37], [39]

The aim of the present study was to address the role of PRL in the regulation of anterior pituitary cell turnover and PRLR expression in the anterior pituitary of cycling females. To that end, we designed a series of *in vivo* experiments involving normal rat/mice as well as two genetically-modified mouse models in which PRLR signaling is down-regulated or totally abolished.

## Materials and Methods

### Drugs and reagents

All drugs, media and supplements were obtained from Sigma (St. Louis, MO, USA) except fetal calf serum (Natocor, Córdoba, Argentina), amphotericin B, essential aminoacids and gentamicin (Invitrogen, Carlsbad, CA, USA), all terminal deoxynucleotidyl transferase-mediated deoxyuridine triphosphate nick end-labeling (TUNEL) reagents (Roche Molecular Biochemicals, Mannheim, Germany) and the reagents described below.

### Animals

Mice and rats were housed in controlled conditions of light (12-hour light-dark cycles) and temperature (20–22°C), and were fed ad libitum.

### Ethical statements

This study was approved by the Comité Régional d'Éthique pour l'Expérimentation Animale, Ile-de-France, Université Paris Descartes (authorization number: P2.VG.120.09) and by the Animal Care Use Committee of the School of Medicine, University of Buenos (CICUAL), University of Buenos Aires, approval ID: Res. (CD) N° 2831/10.

### *In vivo* treatments of female rats

Adult Wistar female rats (200–220 g) were ovariectomized (OVX) under ketamine (100 mg/kg, i.p.) and xylazine (10 mg/kg, i.p.) anesthesia and ketoprofen (5 mg/kg) for analgesia, 2 weeks before treatments.

OVX rats were injected with ovine PRL (oPRL) (1 mg/kg, i.p) or vehicle (NaCl 0.9%). This dose of PRL results in serum concentrations of ∼700 ng/ml at 30 min and ∼500 ng/ml 1 h after i.p. administration [47]. Rats were co-injected with bromodeoxyuridine (BrdU, 50 mg/kg) [24] and euthanized 6 h later.

In other experiments, rats were injected with the dopamine D2 receptor (D2R) antagonist sulpiride (5 mg/kg, i.p.) [48] and BrdU (50 mg/kg) and killed 6 h later.

Anterior pituitary glands were removed within minutes after decapitation. Cells were dispersed and fixed. For this procedure, anterior pituitary glands were washed with Dulbecco Eagle's Modified Medium (DMEM) containing 3 mg/ml bovine serum albumin (DMEM-BSA). Then, anterior pituitaries were cut into small fragments and dispersed enzymatically by successive incubations in DMEM-BSA containing 0.75% trypsin, 10% charcoal-dextran-adsorbed fetal calf serum (FCS) and 45 U/ml deoxyribonuclease type I (DNAse). Finally, the cells were dispersed by extrusion through a Pasteur pipette in Krebs buffer without Ca^2+^ and Mg^2+^. Dispersed cells were washed and resuspended in DMEM with 10% FCS. In all procedures, cell viability as assessed by trypan blue exclusion was over 85%. Cells were fixed using ice-cold 70% ethanol in PBS overnight at −20°C [35].

### PRLRKO mice

To evaluate the role of PRLR signaling in the control of anterior pituitary cell renewal during the estrous cycle, we used PRLRKO mice in which PRLR signaling is totally abolished due to the absence of PRLR expression. These mice were generated on C57BL/6 genetic background as previously described [49]. Two to 3 month-old mice were used, because they do not present pituitary enlargement at this age [49], and consequently have conserved anterior pituitary cell populations. PRLRKO and wild type (WT) littermates cycling female mice were injected with BrdU (50 mg/kg, i.p) 24 h before euthanasia. Animals were euthanized by cervical dislocation at diestrus or proestrus, as determined by daily vaginal smears. Pituitaries were removed within minutes, weighed and processed for TUNEL assay, BrdU incorporation detection and PRL co-immunostaining.

### Transgenic mice expressing the pure PRLR antagonist Δ1–9-G129R-hPRL

To evaluate the role of PRL in the control of PRLR expression, we used transgenic female mice in which systemic expression of the pure PRLR antagonist Δ1–9-G129R-hPRL impairs PRLR signaling (TG^Δ1–9-G129R-hPRL^ mice). These mice were generated on BALB/c-J background as previously described [7]. As young but not old TG female mice have altered estrous cycle [50], we used 12 month-old TG^Δ1–9-G129R-hPRL^ female mice and WT littermates, which were euthanized at proestrus or diestrus, then pituitaries were removed as described above. After neurointermediate lobe removal, anterior pituitaries were processed for RNA extraction.

### Detection of BrdU incorporation by flow cytometry (FACS)

In rats, BrdU incorporation was detected by FACS. After fixation in ice-cold 70% ethanol by gently vortexing, cells were centrifuged and incubated in a solution containing 1% paraformaldehyde (PFA), 0.01% Tween-20, 1 h at 20°C. Then, the cells were incubated with DNAse I 100 U/ml diluted in NaCl 0.15 M, MgCl_2_ 4.2 M pH 5 for 25 min at 37°C, centrifuged and incubated with a fluorescein (FITC)-conjugated anti-BrdU antibody or the corresponding isotype control (BD Bioscience, San Jose, CA, USA) for 40 min at 20°C. Cells were washed with PBS and incubated with 1% PFA for 15 min. After centrifugation, cells were resuspended in PBS until FACS analysis. Fluorescence intensity of ≥6,000 gated cells/tube was analyzed by FACS using a FACScan (BD Bioscience, San Jose, CA, USA). Analysis of BrdU positive cells was performed using WinMDI 98 software.

### Cell-cycle analysis by FACS

After fixation in ice-cold 70% ethanol, anterior pituitary cells from OVX or treated- OVX rats, were centrifuged and DNA was stained with propidium iodide (PI, 50 µg/ml) in PBS containing ribonuclease (10 µg/ml) for 20 min at 37°C [24], [35]. After centrifugation, immunostaining of lactotropes was performed using guinea pig antiserum directed against rat PRL (Dr. A.Parlow, National Hormone and Pituitary Program, Torrance, CA, USA) (1∶2,000, 1 h at 37°C), washed in PBS and then incubated with a FITC-conjugated anti-guinea pig antibody (Chemicon International, Temecula, CA., USA) (1∶75, 40 min at 37°C). For isotype controls, the cells were incubated with guinea pig serum instead of PRL antiserum [51]. Cells were washed, resuspended in PBS and analyzed by FACS. Fluorescence intensity of ≥10,000 gated cells/tube was analyzed using a FACScan.

Cells with a PI staining intensity lower than the G0/G1 peak were considered hypodiploid. Analysis of DNA content and PRL-positive cell determination were performed using WinMDI 98 software. The analysis of hypodiploidy and cell cycle in lactotropes was performed gating the PRL-positive population. Determination of cells in Sub G0/G1 (hypodiploid cells), G0/G1, S and G2/M-phases of the cell cycle, was performed using WinMDI 98 and Cylcherd 1.2 softwares [24], [35].

### TUNEL assay and detection of BrdU incorporation in anterior pituitary sections

Pituitaries from PRLRKO and WT cycling female mice were removed immediately after euthanasia, weighed, fixed in 4% PFA in PBS (pH 7.4) for 4 h and embedded in paraffin. Sections (4 µm) were deparaffinized in xylene and rehydrated in graded ethanol. Antigen retrieval was performed by microwave irradiation. For TUNEL assay, DNA strand breaks were labeled with digoxigenin-deoxyuridine triphosphate using terminal deoxynucleotidyl transferase (0.18 U/µl) according to the manufacturer's protocol. After incubation in PBS with 10% sheep serum for 90 min, sections were incubated with antidigoxygenin- FITC antibody (1∶10) to detect incorporation of nucleotides into the 3′-OH end of damaged DNA [24], [52].

For BrdU incorporation detection, slides were permeabilized with PBS-Triton X-100 0.1% for 17 min at RT. After incubation with 3% BSA in PBS-Triton for 30 min, sections were incubated with anti BrdU antibody in DNAse solution (GE Healthcare, Little Chalfont, Buckinghamshire, UK) according to manufacturer's protocol [24]. Sections were washed and incubated with an anti-mouse FITC secondary antibody (Chemicon International, Temecula, CA., USA) 1∶200 in PBS-Triton 0.5%, with 1% horse serum.

In order to identify TUNEL-positive or BrdU-positive lactotropes, after washing with PBS-Triton 0.5%, slides were incubated with 10% goat serum in PBS-Triton 0.5% for 90 min followed by an anti-mouse PRL antiserum (Dr. A.Parlow, National Hormone and Pituitary Program) 1∶200 in PBS-Triton 0.5% overnight. After washing, slides were incubated with an anti rabbit secondary antibody conjugated with rhodamine (Chemicon). Sections were mounted with Vectashield (Vector Laboratories, Inc., Burlingame, CA, USA) containing 4, 6 diamidino-2-phenylindoledihydrochloride (DAPI) for DNA staining and visualized in a fluorescent light microscope (Axiophot). Since the number of TUNEL-positive or BrdU-positive cells in sections from *in vivo* studies were very low, apoptosis and proliferation were expressed as the number of TUNEL-positive cells/field, BrdU-positive cells/field, TUNEL-positive PRL-positive cells/field (TUNEL-positive lactotropes), BrdU-positive PRL-positive cells/field (BrdU-positive lactotropes) or TUNEL-positive PRL negative cells/field (TUNEL-positive non lactotrope cells) and BrdU-positive PRL negative cells/field (BrdU-positive non lactotrope cells) as previously described [24], [52]. Cells from the neurointermediate lobe were excluded from the count. TUNEL-positive or BrdU-positive anterior pituitary cells were counted in 30–40 fields (x 400) of anterior pituitary sections from each mouse. The mean of TUNEL-positive cells or TUNEL-positive lactotropes/field or BrdU-positive cells or BrdU-positive lactotropes/field from each mouse was considered as an individual value [24], [52].

### RNA extraction and Real-Time PCR

Anterior pituitaries from TG and WT cycling female mice were removed, washed in RNAeasy Solution (Qiagen Inc., Santa Clarita, CA, USA) and immediately frozen in liquid nitrogen. RNA was extracted using RNAeasy Micro Kit (Qiagen) following the manufacturer's protocol. Briefly, frozen anterior pituitaries were homogenized using a cold mortar and pestle and placed in 350 µl of RTL lysis buffer (Qiagen) containing β-mercaptoethanol, re-homogenized with a needle and centrifuged. The supernatant was washed with 70% ethanol, centrifuged, and transferred onto an RNAeasy spin column (Qiagen). RNA was collected with 14 µl RNAse-free water and stored at −80°C. Reverse transcription was performed using SuperScript II Reverse Transcriptase according to the manufacturer's protocol. One hundred twenty five ng of total RNA were reverse transcribed. After incubation for 5 min at 65°C with 1 µl oligo (dT) and 1 µl of dNTP Mix, RNA samples were incubated for 2 min at 42°C with a mix containing DTT and RNAse OUT (Invitrogen). After addition of 1 µl SuperScript II RT (Invitrogen), samples were incubated for 50 min at 42°C and the reaction was heat-inactivated (70°C for 15 min). Finally, samples were treated with RNAse H (Invitrogen) to remove any possible RNA complementary to cDNA.

For Real Time PCR, we used forward primers mapping mouse PRLR extracellular domain (ECD) common to all isoforms, and a reverse primer specific to intracellular domains (ICD) of mouse PRLR_long_, S1, S2 or S3 PRLR_short_ isoforms (Table 1) [24]; cyclophilin was used as a reference as previously described [24], [53]. All primers were obtained from Eurogentec (Liège, Belgium).

**Table 1:** Primers used for real time PCR analysis of PRLR isoform expression.

Primer	Sequence
PRLR ECD Forward	5′-TAAAAGGATTTGATACTCATCTGCTAGAG-3′
PRLR ICD Long Reverse	5′-TGTCATCCACTTCCAAGAACTCC-3′
PRLR ICD S1 Reverse	5′-CATAAAAACTCAGTTGTTGGAATCTTCA-3′
PRLR ICD S2 Reverse	5′-GGAAAAAGACATGGCAGAAACC-3′
PRLR ICD S3 Reverse	5′-AGTTCCCCTTCATTGTCCAGTTT-3′

ECD: extracellular domain; ICD: intracellular domain

Real Time PCR was performed using an Applied Biosystems 7300 Real-Time PCR System. For each reaction, 25 µl of solution containing 5 µl cDNA, 0.25 µl of 20 µM forward and reverse primers and 12.5 µl Power SyberGreen PCR Master Mix (Applied Biosystems, Carlsbad, CA, USA) were used. All reactions were performed in duplicate. Negative controls included amplification of RNA (without reverse transcription) and water. Amplification was initiated by a 2 min pre-incubation at 50°C, followed by 40 cycles at 95°C for 30 s, 60°C for 1 min, 95°C for 15 s, 60°C for 30 s, terminating at 95°C for the last 15 s (melting). We used the 2^−ΔCt^ method to determine the effect of the experimental treatment (presence of the antagonist in transgenic mice or estrous cycle variation) on the expression of the candidate internal control gene. The expression for cyclophilin was not different in WT and TG transgenic mice or between different estrous cycle stages. Validation of the 2^−ΔCt^ method in our experiments was performed by determining that amplification efficiencies of PRLR_long_, PRLR_short_ isoforms and cyclophilin were similar, following the procedure published by Livak and Schmittgen et al [54]. Linearity of real-time RT-PCR signaling was determined with wide-range serial dilutions of reference cDNA and linear correlations were found between the amount of cDNA and the Ct for the duration of at least 40 real-time RT-PCR rounds. Expression levels were normalized to mouse cyclophilin expression, performed in parallel as endogenous control [54]. Real Time-PCR data were analyzed by calculating the 2^−ΔCt^ value for each experimental unit, Ct being the cycle threshold number and ΔCt being the difference between the Ct values for total or each PRLR isoform and cyclophilin [24].

### Determination of PRL serum levels by Radioimmunoassay (RIA)

Serum PRL levels were measured by RIA using reagents provided by the National Institute of Diabetes and Digestive and Kidney Diseases, National Hormone and Pituitary Program (Dr. A. F. Parlow, Torrance, CA). Results were expressed in nanograms per milliliter in terms of rat PRL RP3. Intra- and interassay coefficients of variation were 6.9 and 11.6%, respectively [55].

### Statistical Analysis

Data were expressed as mean ± SEM. The significance of the differences between means was determined by Student's *t* test or two-way ANOVA followed by Tukey's test. Differences were considered significant if p<0.05. Statistical analyses were performed using GraphPad Prism 6 software.

## Results

### Acute PRL injections regulate proliferation and apoptosis of female rat lactotropes

**Figure 1:**
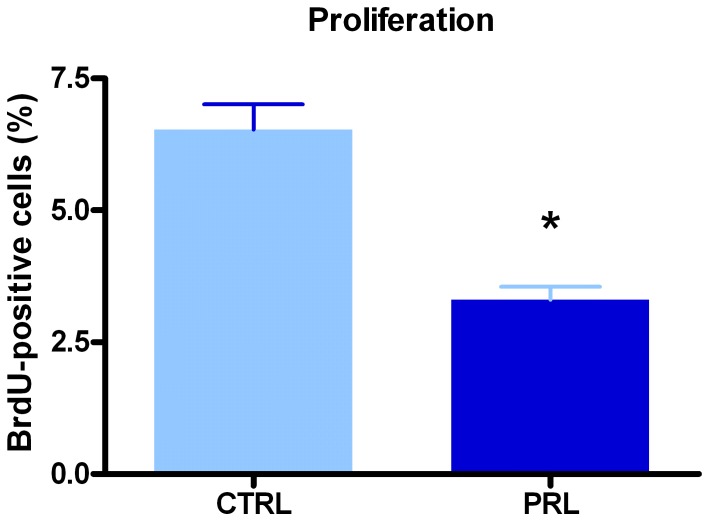
PRL decreases anterior pituitary cell proliferation *in vivo*. Ovariectomized rats (n = 4 rats/group) were injected with oPRL (1 mg/kg, 6 h) or saline and BrdU (50 mg/kg, 6 h). Proliferation rate was determined by the detection of BrdU incorporation and FACS analysis. Each column represents the mean ± SEM of the percentage of BrdU-positive cells. *p<0.05 vs. respective control (CTRL) animals injected with vehicle, Student's *t* test.

**Figure 2:**
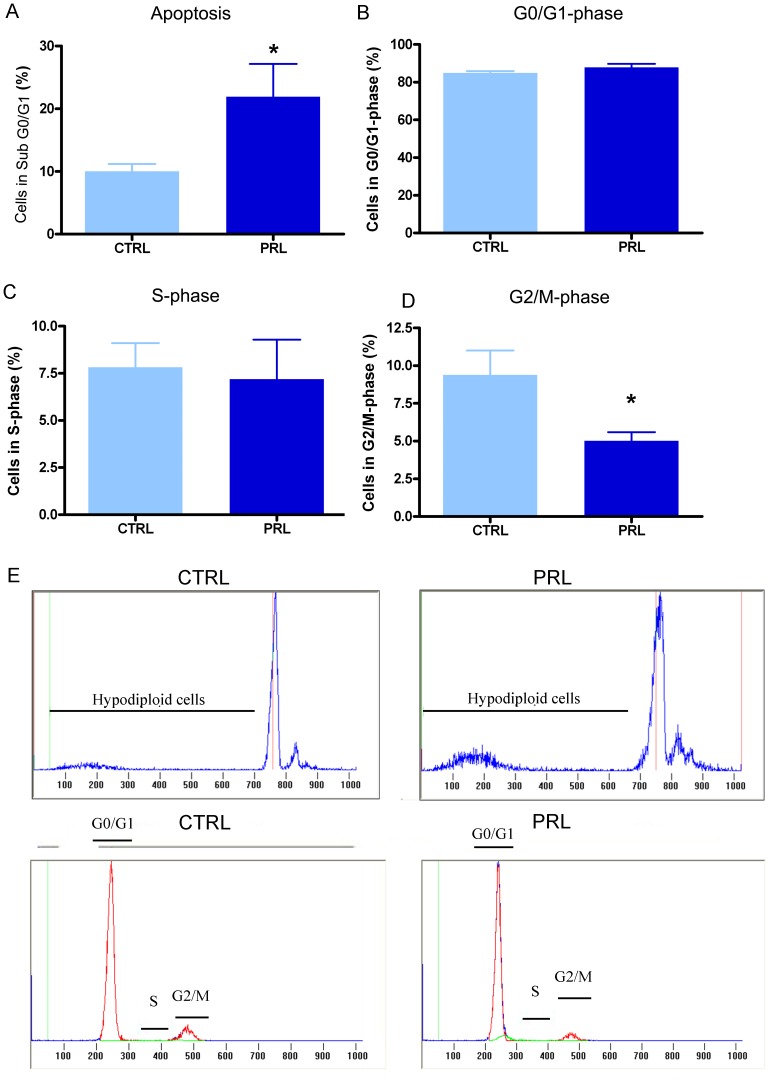
PRL increases anterior pituitary cells apoptosis *in vivo* and decreases the percentage of total anterior pituitary cells in G2/M-phase. Ovariectomized rats (n = 12 rats/group) were injected with oPRL (1 mg/kg, 6 h) or saline. A: Apoptosis was determined as the percentage of hypodiploid cells, using PI and FACS. Each column represents the mean ± SEM of the percentage of sub G0–G1 cells. *p<0.05 vs. respective control animals injected with vehicle, Student's *t* test. B–D: Cell cycle was analyzed by FACS using PI. Each column represents the mean ± SEM of the percentage of cells in G0/G1-phase (B), cells in S-phase (C) and cells in G2/M-phase (D). * p<0.05 vs. control animals injected with vehicle, Student's *t* test. E: Representative histograms showing hypodiploidy or cells in each cell cycle stage in CTRL and oPRL-treated animals.

**Figure 3:**
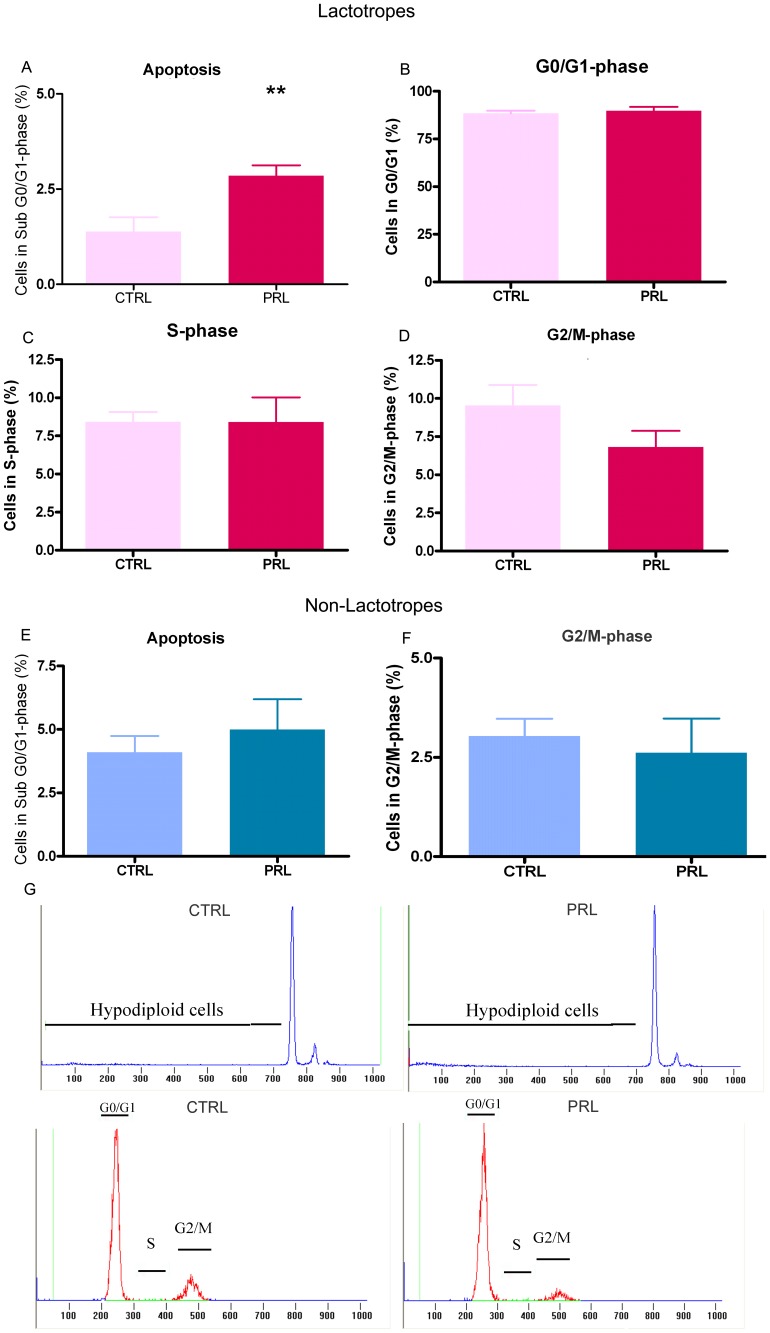
PRL increases lactotrope apoptosis *in vivo*. Lactotropes were identified by PRL immunostaining and analyzed by FACS. Apoptosis and cell cycle analysis were performed in the PRL-positive subpopulation. A: Apoptosis was determined by FACS using PI. Each column represents the mean ± SEM of the percentage of sub cells in G0–G1. *p<0.05 vs. CTRL animals injected with vehicle, Student's *t* test. B–D: Cell cycle was analyzed by FACS using PI. Each column represents the mean ± SEM of the percentage of cells in G0/G1-phase (B), cells in S-phase (C) and cells in G2/M-phase (D) p = 0.05 for G2/M in oPRL respect to CTRL, Student's *t* test. E–F: oPRL treatment did not change the apoptosis rate (E) or the percentage of cells in G2/M in the PRL-negative subpopulation. G: Representative histograms showing hypodiploidy or cells in each stage of the cell cycle in CTRL and PRL-treated animals.

In cycling females, anterior pituitary cells are exposed to high PRL levels during the afternoon of proestrus, the stage of the estrous cycle with the highest rate of apoptosis and the lowest rate of proliferation. Therefore, to examine whether PRL acutely affects proliferation or apoptosis of anterior pituitary cells, OVX rats were injected with oPRL and euthanized 6 h later [47]. Ovariectomy was performed to ensure low and stable plasma levels of endogenous PRL and to avoid effects of gonadal steroids on anterior pituitary cell apoptosis and proliferation [32], [56], [57], [58], [59]. Acute oPRL treatment decreased the proliferation rate of anterior pituitary cells as determined by monitoring BrdU-positive cells (Fig. 1). In contrast, apoptosis was increased by oPRL as determined by the percentage of cells with hypodiploid DNA content (Fig. 2A). Cell-cycle analysis showed that this effect was correlated to a decreased number of cells in G2/M phase, without changes in the percentage of cells in G0/G1 or S-phase (Fig. 2 B–D). To investigate whether PRL specifically regulates lactotrope renewal, we characterized cell cycle progression and hypodiploid content of DNA in cells positively stained with an anti-PRL antibody. Lactotrope apoptosis was higher in rats injected with oPRL than in control OVX animals (Fig. 3A, G). We also observed that oPRL reduced the percentage of lactotropes in G2/M with borderline significance (p = 0.053) (Fig. 3D), without other alteration in the progression of the cell cycle in the lactotrope subpopulation (Fig 3 B, C). The proapoptotic and antiproliferative effects of oPRL were not observed in the non-lactotrope subpopulation (Fig. 3E–F and G).

### Endogenous PRL regulates apoptosis and proliferation of female rat anterior pituitary cells

**Figure 4:**
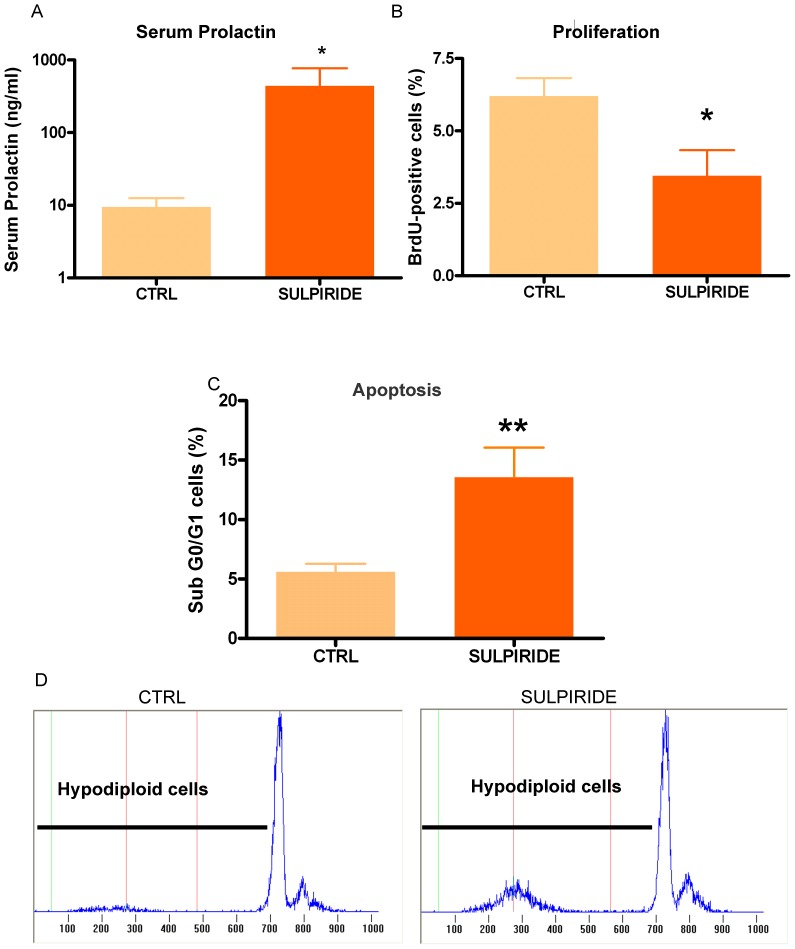
Sulpiride decreases anterior pituitary cell proliferation *in vivo*. Ovariectomized rats (n = 5–7 rats/group) were injected with sulpiride (5 mg/kg, 6 h) or saline, and with BrdU (50 mg/kg, 6 h). A: Sulpiride treatment induced hiperprolactinemia in OVX rats. Each column represents serum PRL levels ± SEM in CTRL or sulpiride-treated animals. *p<0.05 vs. CTRL animals injected with vehicle, Student *t* test. B: Proliferation rate determined by the detection of BrdU incorporation and FACS. Each column represents the mean ± SEM of the percentage of BrdU-positive cells. *p<0.05 vs. CTRL animals injected with vehicle, Student *t* test. C: Apoptosis was determined by FACS, using PI. Each column represents the mean ± SEM of the percentage of sub-G0-G1 cells. **p<0.01 vs. CTRL animals injected with vehicle, Student's *t* test. D: Representative histograms showing hypodiploidy in CTRL and sulpiride-treated animals.

Besides its inhibitory action on PRL secretion, dopamine modulates anterior pituitary cell turnover by inducing apoptosis and decreasing proliferation of lactotropes [34], [60]. Hypothalamic dopaminergic neurons express PRLR, and variations in serum PRL levels induce changes in the dopaminergic tone [60]. Since PRL administration may lead to an increase in dopamine reaching the anterior pituitary [36], dopamine could be involved in changes in anterior pituitary cell turnover induced by PRL injection. To circumvent this possibility, we induced hyperprolactinemia in OVX rats by the administration of sulpiride, a D2R antagonist. As expected, sulpiride administration increased serum PRL levels up to ∼400 ng/ml (Fig. 4A). Similarly to what was observed in rats injected with oPRL, sulpiride administration decreased BrdU incorporation (Fig. 4B) and increased apoptosis of anterior pituitary cells (Fig. 4C). These results suggest that PRL exerts a direct antiproliferative and proapoptotic effect on anterior pituitary cells, independently of dopamine action.

### The absence of PRLR signaling abrogates anterior pituitary cell apoptosis during the estrous cycle

**Figure 5:**
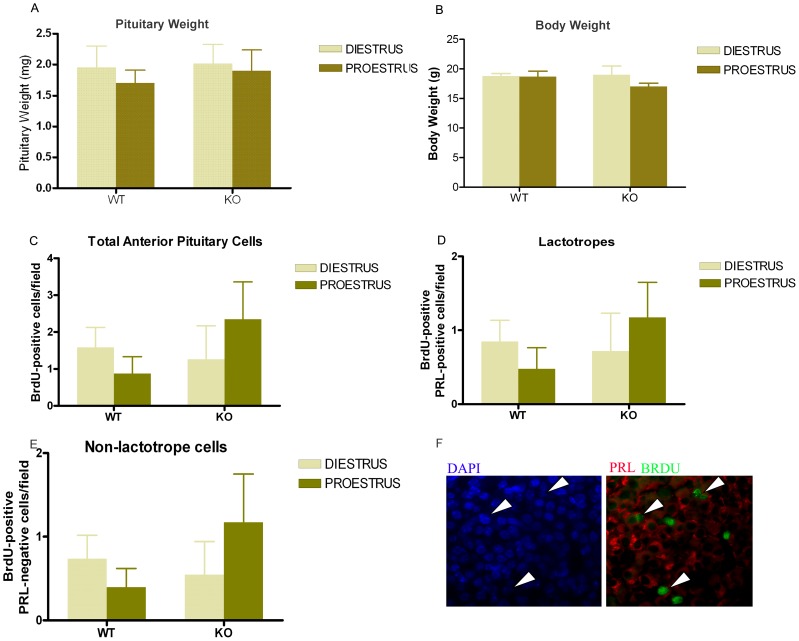
Anterior pituitary cell proliferation in WT and PRLR KO mice at proestrus or diestrus. Wild type and PRLRKO mice (6–10 animals per group) were injected with BrdU (50 mg/kg, 24 h) and sacrificed at proestrus or diestrus. Proliferation was determined by BrdU incorporation in tissue sections. A, B: Body weight (A) and pituitary weight (B) of WT and PRLRKO mice euthanized at diestrus or proestrus, expressed as mean ± SEM, two-way ANOVA. C: Each column represents the media ± SEM of proliferating total anterior pituitary cells (BrdU-positive cells/field). Two-way ANOVA. D: Each column represents the media ± SEM of proliferating lactotropes (BrdU-positive PRL positive cells/field), Two-way ANOVA. E: Each column represents the media ± SEM of proliferating non-lactotrope cells (BrdU-positive PRL-negative cells/field). Two-way ANOVA. F: Representative microphotographs of anterior pituitaries from KO mice euthanized at proestrus. Arrow heads indicate BrdU-positive lactotropes. Arrows indicate BrdU-positive cells.

In both mice [61], [62] and rats [36], serum PRL levels are relatively constant all-over the estrous cycle, except at proestrus, when a sharp increase in PRL secretion occurs in response to high circulating levels of estrogens [36]. At that time, the proliferation index in rats is the lowest. To evaluate if the antiproliferative effect of PRL is implied in the control of the proliferation rate that occurs at proestrus, we studied the effects of PRLR signaling on pituitary cell proliferation in WT and PRLRKO mice sacrificed at diestrus or proestrus. As previously reported, no difference in body or pituitary weight was observed in 2–3 month old PRLRKO mice versus WT littermates (Fig. 5A, B) [23]. Proliferation was assessed by *in vivo* BrdU (24 h) incorporation (Fig. 5). In WT mice, there were no differences in the proliferative index of total anterior pituitary cells, lactotropes or non-lactotrope cells between diestrus and proestrus, although the global trend was a decrease in proestrus (Fig. 5C–E). In PRLRKO mice, we again observed no significant difference of proliferation in the various cell populations, but interestingly the trend was to increase in proestrus, opposite to that seen in WT mice (Fig. 5C–E).

**Figure 6:**
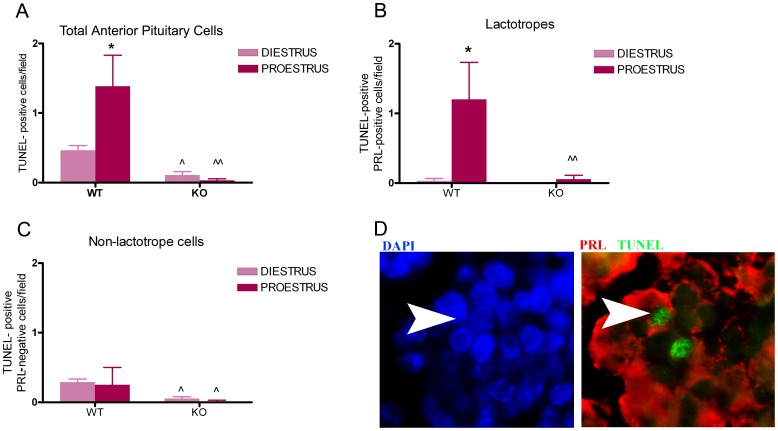
Apoptosis in lactotropes but not in non-lactotrope cells varies during the estrous cycle and decreases in PRLRKO mice. Wild type and PRLRKO mice (6–10 animals per group) were euthanized at proestrus or diestrus. Apoptosis was determined by TUNEL assay in tissue sections. A: Each column represents the media ± SEM of TUNEL-positive cells/field. *p<0.05 vs. diestrus WT, ∧p<0.01 vs. diestrus WT, ∧∧p<0.01 vs. proestrus WT. Two-way ANOVA, followed by Tukey's test. B: Each column represents the media ± SEM of apoptotic lactotropes (TUNEL-positive PRL-positive cells/field). *p<0.05 vs. diestrus, ∧∧p<0.01 vs. proestrus WT. Two-way ANOVA, followed by Tukey's test. C: Each column represents the media ± SEM of apoptotic non-lactotrope cells (TUNEL-positive PRL-negative cells/field). ∧p<0.05 vs. diestrus WT or proestrus WT. Two-way ANOVA, followed by Tukey's test. D: Representative microphotographs of anterior pituitaries from WT mice euthanized at proestrus. Arrow heads indicate a TUNEL-positive lactotrope.

The proestrus peak in circulating levels of PRL coincides with the highest rate of apoptosis in the rat anterior pituitary [25]. To evaluate whether the proapoptotic effect of PRL is involved in anterior pituitary cell renewal, especially at proestrus, we determined the rate of apoptosis in the anterior pituitary gland from WT and PRLRKO mice euthanized at proestrus or diestrus (Fig. 6A–C). In WT mice apoptosis was higher at proestrus than at diestrus (Fig. 6A). When PRLR signaling was totally abolished (i.e. in PRLRKO mice), the number of apoptotic anterior pituitary cells was markedly decreased compared to WT mice both at diestrus and at proestrus (Fig. 6A). In fact, the high apoptotic rate of total anterior pituitary cells and lactotropes normally observed at proestrus was absent in PRLRKO mice (Fig. 6A, 6B). In contrast, no differences in the apoptotic rate of non-lactotrope cells were observed between diestrus and proestrus (Fig. 6C). Taken together, these results suggest that i) the main subpopulation implied in mouse anterior pituitary cell turnover at proestrus is the lactotrope subpopulation, and ii) PRLR signaling is a major regulator of cell apoptosis underlying this process.

### Prolactin regulates expression of its own receptor during the estrous cycle

**Figure 7:**
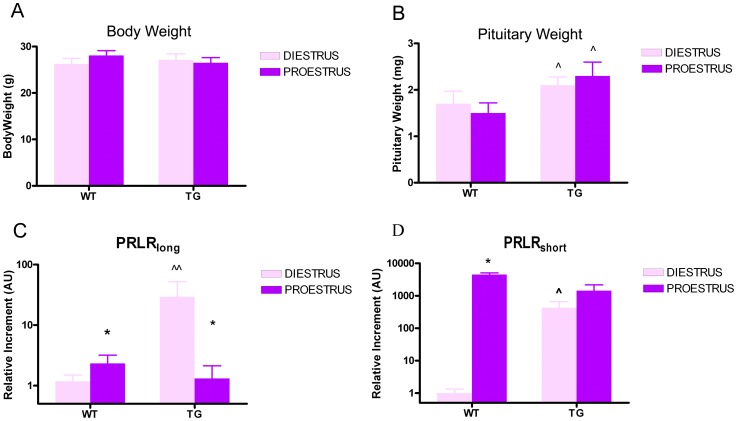
PRLR antagonism increases the expression of the long and short isoforms of PRLR. A, B: Body weight and pituitary weight of WT and TG^Δ1–9-G129R-hPRL^ mice, euthanized at diestrus or proestrus ∧p<0.05 vs. respective WT at diestrus or proestrus. Two-way ANOVA followed by Tukey's test. C, D: Expression of PRLR isoforms in the anterior pituitary from WT and TG^Δ1–9-G129R-hPRL^ mice, euthanized at diestrus or proestrus. Real Time PCR was performed using specific primers for long and short (S1, S2, S3) PRLR isoforms. The S1 short isoform was not detected. Each column represents the relative increment ± SEM with respect to the expression of PRLR_long_ (C) or PRLR_short_ (D) in control animals (WT mice at diestrus) (n≥5 animals/group). *p<0.05 vs. respective diestrus, ∧p<0.0.5 and ∧∧p<0.01 vs. respective WT at diestrus. Two-way ANOVA, followed by Tukey's test.

To further investigate the effects of PRL on the control of pituitary cell homeostasis at proestrus, we determined the expression profile of PRLR isoforms in anterior pituitaries of female mice when apoptosis occurs. To that end, we used transgenic mice expressing the pure PRLR antagonist Δ1-9-G129R-hPRL, which competes with endogenous PRL for PRLR activation [7], [24]. In agreement with previous results involving male mice [24], we observed that chronic blockade of PRLR signaling by the PRLR antagonist resulted in increased pituitary weight in TG^Δ1–9-G129R-hPRL^ female mice compared to WT littermates (Fig. 7B), whereas body weight was unaffected (Fig. 7A).

Using isoform-specific qPCR, we were able to detect three PRLR isoforms in the anterior pituitary from both WT and TG^Δ1–9-G129R-hPRL^ mice (PRLR_long_, PRLR_short_ S3 and, to a much lesser extent, PRLR_short_ S2). The ratios of PRLR_long/_PRLR_short_ expression in the different experimental conditions (WT and TG^Δ1–9-G129R-hPRL^ at proestrus or diestrus) that were analyzed in this study are shown in Table 2. In WT mice, PRLR_long_ is the predominant isoform (36-fold over PRLR_short_) expressed at diestrus. The expression of both PRLR_long_ (Fig. 7C) and PRLR_short_ S3 (Fig. 7D) increased at proestrus, but with a much higher amplitude for the latter; as a result, the ratio of both isoforms dropped to nearly 1∶1. In TG^Δ1–9-G129R-hPRL^ mice, expression of both PRLR_long_ and PRLR_short_ S3 were dramatically increased with respect to WT mice at diestrus (Fig. 7C, 7E), but again PRLR_long_ was very predominant (57-fold). At proestrus, in contrast to WT mice, expression of PRLR_long_ decreased compared to diestrus, while PRLR_short_ S3 expression was unchanged. Taken together, these results show that the expression of PRLR varies during the estrous cycle in an isoform-specific manner, which ultimately affects the long/short isoform ratio. Chronic blockade of PRLR signaling by the antagonist alters the isoform-specific regulation that normally occurs during the estrous cycle, which strongly suggests that PRL modulates the expression of its own receptor in the pituitary.

**Table 2:** PRLR_long_/PRLR_short_ ratios at proestrus or diestrus in WT and TG animals.

WT		TG^Δ1–9-G129R-hPRL^	
DIESTRUS	PROESTRUS	DIESTRUS	PROESTRUS
36.0∶1	1.2∶1	57.0∶1	2.5∶1

## Discussion

The control of cell turnover in the anterior pituitary has been extensively studied by several groups including ours, focusing mainly on the endocrine effect of upstream neurotransmitters and peripheral hormones/cytokines such as dopamine [33], [34], estradiol [31], [32], [51], [63], TNF-α [56], [57], FasL [58], [59], TGF-β [48], [55] or IL-6 [64] to cite only a few. However, the autocrine or paracrine regulation of pituitary cell turnover by pituitary hormones themselves is less well understood. Recently, it has been shown that GH controls somatotrope function via an intracrine mechanism and that deregulation of this process is involved in the development of GH-secreting tumors [65]. In the female anterior pituitary, lactotropes represent up to the 50% of the hormone-secreting cells [36] and are the population with the highest plasticity in the gland, suffering periods of proliferation and apoptosis that are tightly regulated throughout the estrous cycle [10], [25]. Prolactin may act on PRL-producing cells as well as on neighbor, non-lactotrope cells, considering that all of them express the PRLR [11], [12], [21], [41]. In fact, PRL is known to inhibit its own synthesis and secretion at the lactotrope level [46]. Furthermore, we recently observed that PRL controlled anterior pituitary cell turnover in male mice by an autocrine/paracrine mechanism [24]. In contrast to what occurs in males, regulation of pituitary cell functions during the sexual cycle in rodents depends on short-term exposure to hormones. For that reason, we directed our studies to the acute effects of PRL on anterior pituitary cell turnover in order to determine whether this hormone participates in the control of pituitary remodeling. We found that acute hyperprolactinemia, induced either by PRL administration or by dopamine receptor antagonism, reduced cell proliferation and increased apoptosis, indicating that these effects of PRL are dopamine-independent. Also, we found that PRL increased the apoptotic rate and had a non-significant effect on cell proliferation specifically in lactotropes. In other words, PRL acts as an antiproliferative and proapoptotic autocrine/paracrine factor in the pituitary, specifically on lactotrope cells, and this effect occurs after short time stimulation, as may occur during the estrous cycle.

Therefore, we investigated whether the proestrus surge of PRL is involved in the regulation of proliferation and apoptosis of the anterior pituitary gland during the estrous cycle using a mouse model in which PRLR signaling is totally abolished, i.e. the PRLRKO mouse. Wild type animals at diestrus were used as controls, since, at this stage of the cycle, PRL levels are low and, based on previous reports in rats [28], [30], we expected to find basal levels of proliferation or apoptosis.

Although we did not observe significant differences in the proliferation index in PRLRKO mice with respect to WT animals, the sum of our results suggest nevertheless that the peak of PRL also may contribute to lowering the proliferation rate at proestrus. In fact, the lack of a significant effect in the proliferation index in mice during the estrous cycle may be due to the timing of BrdU injection, which was administered 24 hours before euthanasia i.e. the day before the expected proestrus or diestrus. Conversely, we observed that the apoptotic rate in the anterior pituitary from female mice was higher at proestrus with respect to control animals at diestrus, confirming our expectations. The global reduction in the anterior pituitary apoptosis rate, and furthermore the lack of differences in apoptosis between proestrus and diestrus observed in PRLRKO female mice, strongly suggested that PRL acts as a proapoptotic signal during the estrous cycle. These differences in apoptotic rate had no measurable impact on pituitary weight (Fig. 5B). This is not surprising as we at best identified 1 or 2 apoptotic cells per field (Fig. 5C, D), suggesting that apoptosis needs to take place during a certain amount time to eventually impact on pituitary size or weight. This is supported by the fact that pituitary hyperplasia develops in >6 month old PRLRKO mice despite of the fact that significant decrease in apoptosis is already observed from 2–3 months of age (Fig. 5C). Accordingly, variations in apoptotic rate occurring during the estrous cycle (4 days) are probably not sufficient to affect pituitary weight. No attempt was made in the present study to determine the absolute number of pituitary cell in each condition. In summary, this study provides the first evidence that the apoptotic and antiproliferative effects of PRL participates in the regulation of anterior pituitary cell turnover during the estrous cycle.

It has been previously shown that the double PRLRKO/D2RKO mice present more marked pituitary hyperplasia and increased pituitary weight compared to the single D2RKO counterpart [23]. Our results may explain why abrogation of PRLR signaling in PRLKO or PRLRKO mice contributes to pituitary hyperplasia. PRLRKO female mice develop pituitary hyperplasia from 6 months of age, whereas male mice do not show pituitary enlargement until 18 months of age [23]. In females, the apoptotic and proliferative rates are acutely and closely regulated at each stage of the estrous cycle. Our results indicate that in PRLRKO female mice there is a progressive and accumulative effect due to the lack of PRL apoptotic and antiproliferative actions in each proestrus. These results also could explain why PRLRKO female mice develop pituitary hyperplasia earlier than males, although PRL also plays a key role in regulating pituitary remodeling in males [24].

Based on the evidence provided here that PRL, like dopamine, regulates anterior pituitary homeostasis by controlling cell proliferation and apoptosis, our findings further support the earlier hypothesis of Schuff et al [23] that a deficiency of these two individual pathways results in additive effects on the development of pituitary tumors in double PRLRKO/D2RKO mice. In accordance with this hypothesis, we also observed that chronic blockade of PRLR by the antagonist Δ1-9-G129R-hPRL increased the pituitary weight in TG female mice.

**Figure 8:**
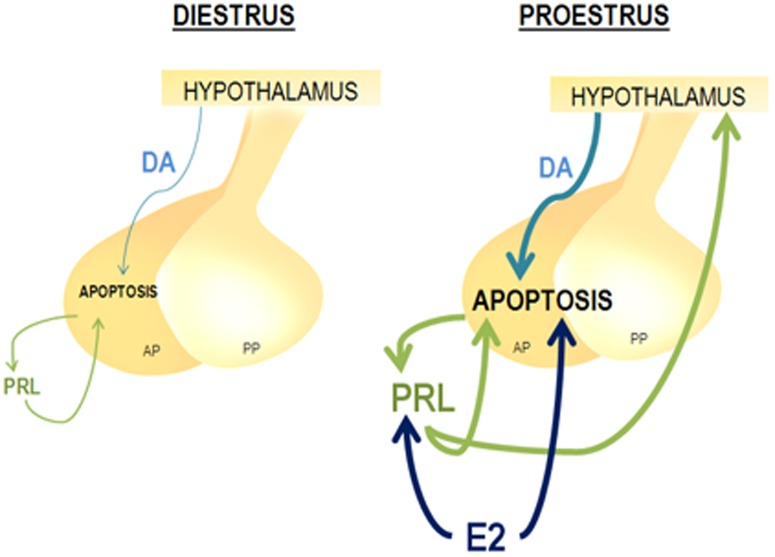
Proposed model of anterior pituitary apoptosis modulation during the estrous cycle. During proestrus the surge of circulating estradiol (E2) (1) increases PRL secretion (2) which, together with E2 itself induces apoptosis of anterior pituitary cells (AP) (3, 3′). PRL stimulates hypothalamic (4) dopamine (DA) release (5) which also contributes to the apoptosis observed in the gland at this stage of the estrous cycle (6). PP: Posterior Pituitary.

Based on these results, we propose a general model depicted in Figure 8. At proestrus, the high circulating levels of estrogens upregulate PRL secretion [36] which in turn, together with estradiol itself [32], [52], induces apoptosis of anterior pituitary cells. Furthermore, PRL stimulates dopamine release from hypothalamic dopaminergic neurons to the portal vessels [66], [67]. Dopamine reaching the anterior pituitary could also contribute to the apoptosis [33], [34] observed in the gland at this stage of the estrous cycle. Estradiol also increases anterior pituitary PRL cleavage into 16 kDa N-terminal fragments, which in turn induce apoptosis in an estrogen-dependent manner [35], by a PRLR-independent mechanism [68].

The molecular mechanisms by which PRL regulates pituitary homeostasis during the estrous cycle are unknown. Since different PRLR isoforms exhibit different capabilities to activate intracellular signaling pathways [4], [37], [38], the ultimate effects of PRL depend not only on the number, but also on the type of receptor isoforms expressed in target tissues. It is thus important to determine the pattern of PRLR expression before moving further in intracellular signaling investigations. In the present study we show that the anterior pituitary from female mice expresses short and long PRLR isoforms and that expression of both are increased at proestrus. This suggests that PRLR expression in the anterior pituitary is regulated by circulating hormones whose levels fluctuate during the estrous cycle. One of them is PRL itself, which may regulate its own functions by modulating the expression of its own receptor. Accordingly, we showed that PRL inhibits expression of its receptor, as PRLR blockade in female mice resulted in increased expression of PRLR_long_ and PRLR_short_ isoforms, which is reminiscent to what we recently reported for male anterior pituitary [24]. It has been previously reported that the main PRLR isoform expressed in anterior pituitaries from prepuberal female mice was PRLR_short_ and that PRL treatment increased the expression of both isoforms of the receptor [44]. The absence of estrous cycle in those young animals could explain the discrepancies with our findings. In another study, metoclopramide-induced hyperprolactinemia was reported to not alter pituitary PRLR expression in either OVX or intact female mice [45]. However, since the latter study did not discriminate PRLR expression at the various stages of the estrous cycle, it is possible that the levels of PRLR that were reported actually represent an average throughout the estrous cycle which may have masked the effect of PRL specifically at proestrus.

At proestrus, the increase of PRLR_short_ expression by far exceeds that of PRLR_long_, resulting in a nearly 1∶1 ratio. The functional outcome and physiological relevance of such a complex regulation of PRLR expression are currently unknown. In fact, the shift from a large excess of PRLR_long_ (diestrus) to similar amounts of long and short isoforms (proestrus) presumably participates in the proapoptotic effect of PRL at proestrus. Although PRL has been shown to be proapoptotic in other tissues or cell types (e.g. keratinocytes, chondrocyte, human myeloma-derived cell lines)[50], the apoptotic signaling mechanism has not been described so far for PRLR. The canonical signaling cascade of the PRLR, namely the Jak2/STAT5 cascade, has been reported in some instance to lead to proapoptotic responses by regulating the expression of Bcl-2 family proteins [38], [69]. However, as PRLR_short_ has been shown to exert dominant negative effect for the activation of Jak2/STAT5 by PRLR_long_ [70], it is unlikely that the increase of PRLR_short_ expression favors such a mechanism to induce apoptosis at proestrus. Clearly, elucidating intracellular mechanisms underlying the proapoptotic effect of PRL at proestrus requires careful investigation during the reproductive cycle using acute PRL stimulation and genetic models as used in this study; this is currently under investigation in our laboratories.

Our results suggest that a failure in PRLR-triggered proapoptotic and antiproliferative effects may play a role in pituitary tumorigenesis. Indeed, it has been reported that human prolactinomas have decreased PRLR expression [71] and that a nonfunctional mutation of the PRLR gene may be related to the presence of microadenomas [72].

In conclusion, our studies show that PRL participates in the control of anterior pituitary cell turnover maintaining pituitary homeostasis throughout the estrous cycle. Hence, we propose that chronic lack of physiological proapoptotic and antiproliferative actions of PRL, and/or changes in PRLR expression, could contribute to alterations in anterior pituitary cell renewal, leading to pituitary hyperplasia and, eventually, tumor development.
